# A Review of Dual-Task Walking Deficits in People with Parkinson's Disease: Motor and Cognitive Contributions, Mechanisms, and Clinical Implications

**DOI:** 10.1155/2012/918719

**Published:** 2011-10-27

**Authors:** Valerie E. Kelly, Alexis J. Eusterbrock, Anne Shumway-Cook

**Affiliations:** Department of Rehabilitation Medicine, University of Washington, 1959 NE Pacific Street, P.O. Box 356490, Seattle, WA 98195-6490, USA

## Abstract

Gait impairments in Parkinson's disease (PD) are exacerbated under dual-task conditions requiring the simultaneous performance of cognitive or motor tasks. Dual-task walking deficits impact functional mobility, which often requires walking while performing concurrent tasks such as talking or carrying an object. The consequences of gait impairments in PD are significant and include increased disability, increased fall risk, and reduced quality of life. However, effective therapeutic interventions for dual-task walking deficits are limited. The goals of this narrative review are to describe dual-task walking deficits in people with PD, to discuss motor and cognitive factors that may contribute to these deficits, to review potential mechanisms underlying dual-task deficits, and to discuss the effect of therapeutic interventions on dual-task walking deficits in persons with PD.

## 1. Introduction

Gait impairments and walking limitations are common among people with Parkinson's disease (PD). While gait abnormalities are not pronounced in the early stages of PD, their prevalence and severity increase with disease progression. Within 3 years of diagnosis, over 85% of people with clinically probable PD develop gait problems [1]. The potential consequences of gait impairments in PD are significant and include increased disability [2, 3], increased risk for falls, and reduced quality of life. Falls are common among people with PD and can result in fear of falling, injury, and hospitalization [4–10]. The estimated prevalence of falls in PD ranges from 40 to 90% and increases with the duration of follow-up [4, 5, 11–16]. It is estimated that 45–50% of falls in this population occur when walking [5, 17], with balance and walking deficits commonly identified as risk factors for falls [5, 10–12, 14, 18, 19]. Reduced quality of life is also associated with balance and gait abnormalities in PD, including festination and freezing of gait [2, 20–24]. In fact, people with PD consider mobility and walking limitations to be among the worst aspects of the disease [25].

Mobility in daily life frequently requires walking while performing simultaneous cognitive or motor tasks, such as talking with a friend or carrying a cup of coffee. Gait impairments in people with PD are exacerbated under such dual-task conditions. In recent years, dual-task walking research has expanded rapidly. The association of gait impairments with adverse consequences like increased fall risk has motivated research into clinical strategies to assess and treat dual-task walking deficits in PD. Several recent review papers have been published on dual-task posture and gait deficits among older adults and in a general neurologic population [26–29], but none have focused specifically on people with PD. While people with PD demonstrate dual-task deficits in a variety of movements, including postural control tasks [30, 31], upper extremity movements [32, 33], and speech [34], the focus of this paper is dual-task walking. The goals of this review are to describe dual-task walking deficits in people with PD, to discuss motor and cognitive factors that may contribute to these deficits, to review potential mechanisms underlying dual-task interference, and to discuss the effect of therapeutic interventions on dual-task walking deficits in people with PD.

## 2. Dual-Task Walking Deficits in PD

Single-task gait impairments in PD include reduced speed and stride length and increased double limb support time and stride-to-stride variability [35–38]. With progression of PD, gait abnormalities worsen, and festination, freezing, and dystonic or dyskinetic gait patterns can emerge [39]. Gait impairments in PD are exacerbated under dual-task conditions, with further reductions in gait speed and stride length [40–46], decreased symmetry and coordination between left and right steps [47, 48], and increased stride-to-stride variability [45, 49, 50]. This section will review reported dual-task walking deficits in people with PD and will consider factors that influence the magnitude of these deficits.

### 2.1. Individual, Task, and Environment Framework

Table 1 summarizes dual-task walking studies in people with PD, including relevant individual, task, and environmental characteristics of each study. Comparing dual-task walking deficits across studies is challenging because of variations in methodology. In Table 1, decrements in walking under dual-task conditions are expressed as a percentage of single-task performance, commonly referred to as the dual-task cost (DTC = [dual-task – single-task]/single-task ∗ 100) [51, 52]. The DTC allows a more direct comparison of dual-task deficits across studies and provides a way to assess the relative effects of individual, task, and environmental factors. For example, a study by Plotnik and colleagues measured gait speed DTCs of 17% in people with moderate PD, on medication, when walking approximately 80 m and performing serial-3 subtractions [45]. Lord and colleagues measured gait speed DTCs of 32% in people with moderate PD, off medication, when walking approximately 6.5 m in their home while carrying a tray and counting auditory tones [43]. Dual-task walking deficits can be compared using the DTC even though these studies varied in terms of the participants' medication status, the concurrent tasks used, and the environment where walking occurred. Because multiple factors differed between studies, it is not clear whether the greater DTCs reported by Lord and colleagues are due to off-medication status, more challenging concurrent tasks, or a more complex home environment. When assessing dual-task deficits in PD, it is important to consider individual characteristics such as the severity of motor and cognitive impairments, the complexity of both walking and concurrent tasks, and the overall challenge presented by the environment. 

### 2.2. Individual Factors

Studies of dual-task walking in PD vary substantially with respect to participant characteristics. Dual-task walking deficits increase with age among healthy adults [29, 60, 61], but people with PD consistently demonstrate greater dual-task walking deficits than healthy, age-matched individuals [42, 44, 50, 54, 59]. For example, O'Shea and colleagues found that people with PD had greater dual-task declines in gait speed than healthy older adults, with gait speed DTCs of −18% to −19% in the PD group compared to −7% in the control group [44]. Most research has examined people with mild-to-moderate disease severity, as measured by the Unified Parkinson Disease Rating Scale (UPDRS) and Hoehn and Yahr scores, although disease severity is associated with dual-task walking deficits [43, 57]. The majority of studies examined the impact of concurrent task performance during the on-medication state, though a small number of studies examined dual-task walking in people with PD in the off-medication state only [43, 59]. Studies that examined the effects of medication demonstrated improvements in dual-task walking performance on-medication compared to off-medication [53, 57]. Some studies specifically examined individuals with PD and freezing [53, 55, 59], motor response fluctuations [45], or a history of falls [62]. For example, research comparing people with PD and freezing to those without freezing demonstrated increased dual-task walking deficits when walking forwards, turning, and walking backwards [53, 55, 59].

### 2.3. Task Factors

Dual-task studies in PD also vary in terms of walking and concurrent task characteristics. Most examined walking on a level surface at a self-selected speed, but some included more complex walking tasks. For example, some walking tasks involved sit-to-stand transfers and/or turning [43, 46, 53, 54, 57–59], and one study examined backwards walking [55]. Concurrent tasks varied in terms of type (cognitive or motor), domain, and difficulty. Concurrent cognitive tasks included mental tracking, such as attentional tasks [43, 50, 59] or arithmetic calculations [41, 42, 44, 45, 47–50, 55, 56], verbal fluency or conversational tasks [42, 53, 54], and memory tasks [46, 50]. Concurrent motor tasks were used less commonly and included carrying objects [40, 43, 46, 57, 58] or manipulating objects [42, 44]. It is not clear whether motor or cognitive tasks have a greater impact on walking in people with PD. One study found similar impacts of cognitive and motor tasks [44], while other studies showed a greater impact of cognitive tasks [42, 46]. However, the tasks incorporated differed in terms of both type and complexity, limiting the ability to make direct comparisons. Studies that controlled task domain and varied task difficulty suggest that more complex tasks have a greater effect on walking in PD [40, 54, 56].

Typically, no specific instructions are provided regarding which task to prioritize during dual-task conditions. In most cases, participants were either instructed to focus on both tasks or instructions were not specified. However, most studies quantified dual-task changes in walking only and did not measure concurrent task performance, making it difficult to determine if there were between-task trade-offs. DTCs provide a means to assess trade-offs between walking and concurrent task performance [63]. In studies that examined dual-task changes in both walking and the concurrent task, most showed declines in both [44, 50]. Only one study demonstrated concurrent task improvements and walking declines under dual-task conditions [42], consistent with trade-offs between tasks and prioritization of the concurrent task over walking.

### 2.4. Environmental Factors

Studies that systematically manipulate environmental factors to determine the effects on dual-task walking deficits in PD are lacking. Most research was conducted in a clinical or laboratory environment, but some was conducted in participants' homes [43, 46, 57, 58]. Studies conducted in the home environment may be more representative of mobility challenges in daily life.

In summary, the literature as a whole confirms the presence of significant dual-task walking deficits among persons with PD, despite methodological variations in participant characteristics, task demands, and environmental constraints. The extent of these deficits appears to vary as a function of individual, task, and environmental characteristics, but the relative contribution of each factor is not well understood. Carefully controlled studies are needed to better quantify how these factors impact dual-task walking deficits in people with PD.

## 3. Motor and Cognitive Factors Contributing to Dual-Task Walking Deficits

### 3.1. Motor Factors

It is not clear how motor and cognitive symptoms contribute to either single-task or dual-task walking deficits in PD. The motor phenotype of PD is heterogeneous, with cardinal features of rigidity, tremor, and bradykinesia [64]. These symptoms, as well as primary impairments in locomotor control pathways [65], can contribute to both single- and dual-task gait abnormalities. The relative contributions of these factors may vary with disease progression. Cardinal symptoms may contribute more to walking deficits early in the disease, while primary gait impairments might predominate later in the disease.

Single-task walking deficits have been associated with a variety of motor symptoms in PD. For example, increased axial rigidity is associated with poorer performance on single-task measures of balance and functional mobility [66, 67]. In addition, rigidity may contribute to reduced lower extremity joint excursions and a forward flexed posture when walking [39]. Bradykinesia can lead to shortened step length and reduced gait speed during walking [39]. Postural instability, another common motor symptom, may contribute to gait impairments such as increased stride-to-stride variability and double limb support.

Several motor factors are associated with dual-task walking deficits in PD. Dual-task gait speed has been associated with disease severity, as measured by Hoehn and Yahr stage [46] and UPDRS motor subscale scores [43]. The severity of PD motor symptoms has also been related to single- and dual-task gait variability both off and on medication [57]. Dual-task walking performance in people with PD has been associated with performance-based measures of balance [46]. Though not a specific motor symptom of PD, some [46], but not all [43, 57], studies have found associations between physical fatigue and dual-task walking deficits in PD. Dual-task walking deficits in PD are also associated with primary gait deficits. Dual-task changes in speed and stride length were associated with performance on single-task mobility tests in people with PD [45]. In addition, dual-task walking deficits were greater in people with PD and freezing of gait compared to those without freezing [53, 55, 59]. Although dual-task walking deficits have been associated with both motor symptom severity and primary gait impairments, the relative contribution of each to dual-task walking deficits has not been well quantified.

### 3.2. Cognitive Factors

PD is associated with a variety of cognitive impairments, including executive function, attention, memory, language, and visuospatial impairments [68–70], that could contribute to dual-task walking deficits. Cognitive profiles in PD are variable [71] and range from mild deficits in specific cognitive domains to severe dementia affecting multiple domains. It is estimated that 19–30% of people with early, newly-diagnosed PD present with cognitive impairments [72–74], and these impairments worsen with disease progression [69]. The presence of mild cognitive impairment in people with PD is associated with development of dementia within 4 years [75]. The prevalence of dementia in PD is estimated at 26–44% [76, 77], with over 80% of people developing dementia within 20 years of diagnosis [13]. Depression can exacerbate cognitive impairments in PD [78], and the frequency of depression in PD is estimated at 25–33% [79, 80].

Specific cognitive functions, such as set shifting, divided or alternating attention, and response inhibition, may be particularly relevant to dual-task walking [28]. Dual-task walking deficits in PD have been associated with impairments in executive function, set-shifting, and attention [43, 45, 46]. For example, Plotnik and colleagues [45] demonstrated a relationship between set shifting, as measured by the Trail Making Test, and dual-task changes in gait speed and step length. Dual-task changes in gait variability were related to executive function, including set shifting and global cognition [45, 50, 57]. Executive function, measured by the Brixton test, has also been associated with gait speed [46] and gait speed DTCs [43]. Deficits in attention were associated with greater deficits in gait variability [57] and increased gait speed DTCs [43]. Finally, depression has been related to gait speed declines and gait variability increases under dual-task conditions in some studies [46, 57], though associations between dual-task parameters and affect (both depression and anxiety) were not supported by all studies [45].

Cognitive impairments can contribute to dual-task walking deficits in various ways. First, they may limit the ability to compensate for gait impairments using cognitive strategies. People with PD are often taught conscious strategies to improve their gait pattern, such as focusing on walking with longer steps. The type and severity of cognitive impairments may limit the ability to use such strategies to compensate for gait abnormalities. Also, impaired executive function might result in the inappropriate or unsafe prioritization of tasks when walking under dual-task conditions. Bloem and colleagues have proposed that increased fall risk in people with PD may result in part from a “posture second” prioritization strategy, in which concurrent tasks are prioritized above walking [81, 82]. Consistent with this idea, falls in PD have been associated with reduced performance on a variety of cognitive measures [83, 84]. The prevalence of cognitive impairments in PD and their associations with dual-task walking deficits suggest that they are an important contributing factor. Further research is needed, however, because little is known about how the domains and severity of cognitive impairments affect dual-task walking deficits and their response to therapeutic interventions.

## 4. Potential Mechanisms Underlying Dual-Task Walking Deficits

The mechanisms responsible for interference between walking and concurrent cognitive or motor tasks in people with PD are not clear. Because multiple factors contribute to dual-task walking deficits, it is likely that a number of different mechanisms contribute to these deficits. In addition, characteristics of the concurrent task, such as type, domain, and difficulty, will impact the mechanisms and resources involved in dual-task performance. This section will review both nonspecific mechanisms proposed to explain dual-task interference across populations as well as specific mechanisms that may contribute to dual-task walking deficits in PD.

### 4.1. Nonspecific Mechanisms

Two general theoretical frameworks have been proposed to explain dual-task interference. Capacity theory conceptualizes the information processing needed for dual-task performance as a flexible but limited resource [27, 85, 86]. Performance of any given task, like walking, requires some portion of this capacity. When two tasks are performed concurrently, competition for limited resources results in dual-task interference and deterioration in performance of one or both tasks [26]. According to this theory, information processing resources such as attention can be flexibly allocated between tasks, with many factors potentially influencing resource allocation [86]. For example, differences in dual-task performance can result from individual differences in overall capacity, and intra-individual variability in dual-task performance can arise from transient variations in effective capacity due to factors like motivation, fatigue, or arousal [86]. Task-related factors also influence resource allocation. For example, a recent meta-analysis demonstrated that dual-task gait speed declines varied as a function of the concurrent cognitive task in healthy young and older adults and a general neurologic population [29].

A second general theory to explain dual-task interference is the bottleneck theory [87]. According to this theory, dual-task performance requires serial or sequential processing of the two concurrent tasks. Dual-task interference results when two tasks compete for the same processing resources. In order to complete one task, processing of the second task is temporarily postponed, resulting in performance decrements in the second task. Dual-task walking studies are limited in their ability to discriminate between these two theories, but these general mechanisms may inform methodological choices and subsequent interpretations.

### 4.2. PD-Specific Mechanisms

Several mechanisms specific to PD may also contribute to dual-task walking deficits. These mechanisms are not mutually exclusive, but might overlap with one another. Consistent with the capacity theory, a first specific mechanism in people with PD is reduced movement automaticity. Automaticity refers to the ability to perform a skilled movement without conscious or executive control or attention directed toward the movement [88, 89]. The control of standing and walking was previously thought to be automatic, but the role of cognitive and executive functions in postural control is increasingly appreciated [26, 28]. For example, in healthy young and older adults, simple reaction times increased when walking compared to sitting, reflecting greater attentional demands for walking [90, 91]. The basal ganglia are proposed to play a role in the automatic control of movement [65]. In people with PD, basal ganglia dysfunction may lead to reduced movement automaticity and the need for increased reliance on cognitive resources to control movements. During dual-task upper extremity movements, people with PD demonstrated greater levels of activity in premotor and prefrontal cortical areas compared to healthy individuals, as measured by functional magnetic resonance imaging [92]. Similarly, people with PD may rely on greater cognitive control during walking, even under single-task conditions [37, 93]. If reduced movement automaticity contributes to dual-task walking deficits in people with PD, rehabilitation strategies designed to improve the automatic control of walking should improve dual-task walking.

A second mechanism that could contribute to dual-task walking deficits in PD is dopamine-mediated dysfunction of the basal ganglia. Multiple parallel pathways through the basal ganglia subserve different functions, including motor, cognitive, and limbic functions [94–96]. Degeneration of dopaminergic neurons in PD appears to affect both motor and cognitive circuits within the basal ganglia. Pathology of basal ganglia circuits that project to the dorsolateral prefrontal cortex may contribute to the executive function deficits that are prominent in people with PD [97, 98]. For example, specific deficits in set shifting, which are associated with dual-task walking deficits in PD [45], are thought to be mediated by the dorsolateral prefrontal cortex [98]. Dual-task walking deficits are improved by anti-parkinson medications [53, 57], supporting the idea that motor and cognitive impairments are due in part to dopaminergic pathways. However, the impact of anti-parkinson medications may be limited to those impairments mediated by dopamine dysfunction, and many studies demonstrate dual-task walking deficits in people with PD in the on-medication state.

A third mechanism that could contribute to dual-task walking deficits in PD is the presence of nondopaminergic pathology, which may affect both gait and cognition. It is increasingly appreciated that the pathology of PD is not limited to dopamine but includes other neurotransmitter systems, such as serotonin, norepinephrine (noradrenaline), or acetylcholine [71, 99, 100]. Dysfunction in multiple neurotransmitter systems may contribute to gait [101, 102] and cognitive impairments in PD [71]. Thus, non-dopaminergic pathways may also contribute to dual-task walking deficits in PD. Consistent with this idea, dual-task walking deficits persist even when people with PD are optimally medicated [42, 44, 50, 54, 59].

In summary, research suggests a number of general and specific mechanisms that may contribute to dual-task walking deficits in PD. These mechanisms are not mutually exclusive, and the relative contribution of each may depend on factors like the symptom profile of the individual and the specific task combination performed under dual-task conditions. A better understanding of the mechanisms responsible for dual-task walking deficits in PD can inform novel therapeutic approaches and enhance our ability to identify optimal interventions.

## 5. Therapeutic Interventions: Impact on Dual-Task Walking Deficits

The effects of various interventions on single-task walking in PD have been well described, but there is less research examining the efficacy of different pharmacological, surgical, or rehabilitative therapies on dual-task walking in this population. Because gait impairments in PD are exacerbated by dual-task conditions, which are common in daily life, it is important to understand how various therapeutic interventions affect dual-task walking.

### 5.1. Pharmacological Interventions

The reported effects of anti-parkinson medications on walking in PD are variable, even under single-task conditions. Medications improve aspects of single-task walking, including gait speed and stride length, but may not influence others, like stride-to-stride variability [38, 103, 104], festination, and freezing of gait [39, 105, 106]. As noted above, anti-parkinson medications increase speed and decrease variability during dual-task walking in PD [57] and even increase dual-task walking speed in those with freezing [53]. Neither of the above studies examined the effects of medication on concurrent task performance, so it is unclear if medication-related improvements were due to trade-offs between walking and the concurrent task. Medications can have limited or adverse effects on cognitive functions like set shifting [107] and certain types of learning [108, 109] that are critical to dual-task walking. As a result, medications could negatively affect dual-task walking or result in dual-task walking improvements at the expense of concurrent cognitive task performance. The positive effects of anti-parkinson medications on dual-task walking are consistent with a contribution from dopaminergic mechanisms, but persistent deficits in the on-medication state suggest that non-dopaminergic mechanisms may also contribute to dual-task interference.

### 5.2. Surgical Interventions

The reported effects of surgery on single-task walking are inconsistent. For example, initial improvements in postural control and gait as a result of deep brain stimulation are not sustained beyond 2–9 years [110]. In the short term, subthalamic nucleus stimulation can improve single-task gait speed and stride length, particularly in the off-medication condition [111, 112], but the individual response to subthalamic nucleus stimulation in the on-medication state is variable [113]. To date, no research has examined the effects of deep brain stimulation or ablative surgeries on dual-task walking in people with PD. The limited research on dual-task upper extremity movements is equivocal, with one study showing no effect of subthalamic nucleus stimulation [114] and one showing a decline [115].

### 5.3. Rehabilitation Interventions

There is considerable research demonstrating training-related improvements in single-task walking in persons with PD [116–122]. However, it is not clear whether dual-task walking deficits can be improved with practice in PD or, alternatively, whether clinicians should teach people with PD to avoid dual-task conditions to improve safety [123]. A variety of rehabilitation strategies to improve dual-task walking in PD have been studied, with most research focusing on external cues, cognitive or attentional strategies, and dual-task gait training.

External visual, auditory, or somatosensory cues improve both single- and dual-task walking in PD [42, 124–129], even among those with de novo PD [130] or cognitive impairment [131]. For example, Rochester and colleagues examined the effects of external rhythmic cues (auditory, visual, and somatosensory) on walking in people with PD [128]. Cueing therapy was provided over nine 30-minute sessions in the home and consisted of training during single- and dual-task walking and during various functional walking tasks. Speed and step length improved during both single- and dual-task cued walking conditions. These improvements transferred to noncued walking and were retained at 6-week follow-up testing. The authors suggest that dual-task walking improvements were likely due to improved walking automaticity. Based on this research, external cueing appears to improve walking under both single- and dual-task conditions in people with PD. However, studies of cue training vary in terms of cueing modality, training duration, tests used for outcomes assessment, and length of follow-up. Further research is needed to determine the parameters of cue training that provide the greatest and most sustained benefits for dual-task walking in PD.

Cognitive or attentional strategies (e.g., focusing attention on walking with long steps) can also improve walking in people with PD [125, 126, 132], but evidence for the efficacy of cognitive strategies to improve dual-task walking is mixed. Dual-task conditions introduce a concurrent task requiring cognitive control. As suggested by the capacity theory of dual-task interference, the need to direct cognitive resources to the concurrent task may limit the ability to use conscious or unconscious cognitive control to improve walking in PD. Some studies indicate that attention can improve dual-task walking [125], while others find that attentional strategies are not effective under dual-task conditions [133]. 

Recent intervention studies have combined dual-task gait training with cognitive strategies to direct attentional focus and task prioritization. Even people with early PD report the need to monitor and consciously correct walking deficits [93]. However, research suggests that people with PD prioritize concurrent tasks over postural tasks under dual task conditions, thereby decreasing safety and increasing fall risk [82]. A number of intervention studies have examined the effects of dual-task training with various instructions regarding task prioritization. Training with instructions to prioritize walking improved gait velocity and stride length under both single- and dual-task conditions [125, 134], with retention at 30 minutes [134]. Dual-task training with instructions to divide attention equally between walking and the concurrent cognitive task also improved dual-task gait speed and stride length, with retention at 30 minutes [135]. However, the same concurrent task was used for both training and outcomes measurement in this study, so it is not clear if these training-related improvements generalize to other dual-task combinations. Canning and colleagues also examined multitask training with divided attention instructions [136]. In this study, the concurrent tasks used during training differed from those used for outcomes measurement. Training improved gait speed and cadence, with improvements retained at 3-week follow-up. Finally, Brauer and Morris examined the effects of dual-task training using variable-priority instructions, where prioritization is shifted between walking and the concurrent task [137]. Gait speed and step length improved for both the trained dual-task combinations and on novel dual-task walking combinations. Performance on the concurrent tasks did not decline, indicating that dual-task walking improvements were not due to between-task trade-offs. The authors suggest that practice may reduce the attentional demands of walking and increase automaticity, thus enabling individuals with PD to attend to more challenging concurrent tasks. Together, these studies suggest that dual-task gait training is an effective intervention, but the relative impact of different instructional sets requires further research. 

One of the limitations in the research on dual-task walking interventions is the lack of consistent and validated measures of dual-task walking performance. Appropriate outcome measures are necessary to determine if a person with PD has dual-task walking deficits and if a given intervention effectively improves these deficits. A variety of tests, including the Stops Walking When Talking test or the Walking and Remembering Test, have been used to assess dual-task walking performance in older adults [138–144]. Few of these measures have been examined in the PD population [54, 81, 145], thus the psychometric properties of these tests in PD are unclear. Future research is needed to determine reliable, valid, and sensitive outcome measures to evaluate dual-task walking performance in people with PD and quantify the response to different interventions.

Research supports the efficacy of rehabilitative interventions, including external cueing, cognitive strategies, and dual-task gait training, to improve dual-task walking deficits in PD. Emerging research is examining additional treatment approaches to improve dual-task walking. For example, treadmill training with virtual reality, designed to incorporate more complex task and environmental conditions, has been shown to improve both single- and dual-task walking in people with PD [146]. Future research is needed to examine optimal treatment parameters for both established and novel dual-task walking interventions, the relative efficacy of different interventions, whether dual-task walking improvements generalize to novel dual-task combinations, and the degree to which improvements in dual-task walking are retained.

## 6. Summary

This paper has reviewed basic and applied research related to dual-task walking deficits in people with PD. Gait impairments under both single-task and dual-task conditions are prevalent in people with PD and are associated with serious consequences. The severity of dual-task walking deficits appears to vary as a function of individual, task, and environmental characteristics, though the relative impacts of each factor are not well understood. Both motor and cognitive impairments have been associated with dual-task walking deficits in persons with PD. However, because the clinical profile of PD is heterogeneous, further research is needed to elucidate the relative contributions of each of these impairments to dual-task walking deficits. A number of general and specific mechanisms may underlie dual-task walking deficits in PD. The role of each is not clear, but might depend on the dual-task combination performed. These mechanisms inform a number of therapeutic interventions. Rehabilitation interventions, including external cues, cognitive strategies, and dual-task gait training, appear to be effective in reducing dual-task walking deficits in PD. However, a better understanding of the individual, task, and environmental factors that influence dual-task walking deficits is critical to refine existing interventions and identify novel therapeutic approaches.

## Figures and Tables

**Table 1:** Summary of studies examining dual-task walking in people with PD. Relevant individual, task, and environmental aspects of each study are included. Dual-task costs for walking and the concurrent task are included where they could be calculated.

	Individual Characteristics			Task and Environmental Characteristics			Results	
Study (PD sample)	Age (yrs)	Disease severity	Cog.	Walking task & environment	Concurrent task	Instruct.	Walking DTC	Concurrent task DTC
Bond and Morris, 2000 [40] (*n* = 12)	65 (10)	Webster score: 13 (5)	STMS: 30 (4)	Walk 10 m (comfortable pace)	Motor: (1) carry tray; (2) carry tray with empty glasses	No prioritization	Speed: (1) −2%; (2) −11% Stride length: (1) +1%; (2) −1% DLS: (1) −2%; (2) −2%	—

Brown et al., 2009 [41] (*n* = 10)	67 (7)	UPDRS: 28 (2) H&Y: 2.3 (0.3)	MMSE: ≥26	Walk 10 m (self- selected pace, unobstructed walkway)	Cognitive: serial-3 subtraction (data presented for no music trials)	None provided	Speed: −20% Stride length: −6% DLS: −12%	—

Camicioli et al., 1998 [53] (*n* = 10)	67 (9) Off-med On-med	UPDRS: 15 (4) UPDRS: 12 (6)	MMSE: 27 (1)	Walk 4.6 m, turn 180°, walk 4.6 m (self-selected pace)	Cognitive: verbal fluency (recite male names)	Not specified	Steps: −40% Time: −50% Steps: −16% Time: −31%	— —

Campbell et al., 2003 [54] (*n* = 9)	74 (7)	H&Y: 2.8 (0.8)	—	Timed Up and Go (3 m; comfortable pace)	Cognitive: (1) repeat phrase; (2) repeat days of the week backwards	Not specified	Steps: (1) +1%; (2) −13% Time: (1) −1%; (2) −31%	—

Galletly and Brauer, 2005 [42] (*n* = 16)	65 (10)	UPDRS: 14 (6)	MMSE: 28 (3)	Walk 10 m (comfortable pace)	Motor: (1) button press Cognitive: (2) serial-3 subtraction; (3) verbal fluency	“Concentrate on both tasks”	Speed: (1) −7%; (2) −21%; (3) −21% Stride length: (1) −4%; (2) −16%; (3) −16%	Motor: (1) +16% Cognitive: (2) +31%; (3) +73%

Hackney and Earhart, 2009 [55] (*n* = 78)	65 (10)	UPDRS: 28 (9)	—	Walk 5 m (comfortable pace) both forward and backward	Cognitive: mental arithmetic (serial-3, 4, and 6 subtraction)	Not specified	*Speed: −33% *Stride length: −14%	—

Hausdorff et al., 2003 [49] (*n* = 10)	Range: 52–82	UPDRS: 14 H&Y: 3.1	MMSE: 27	Walk 20 m (normal pace; level ground)	Cognitive: serial-7 subtraction	“Walk while performing subtractions”	Stride time: −10% Stride time variability: −154%	—

LaPointe et al., 2010 [56] (*n* = 25)	67	H&Y: 2.4 (7)	DRS-2: 136 (7)	Walk 4.3 m	Cognitive: (1) count by 1's; (2) serial-3 subtraction; (3) recite alpha-numeric sequence	Not specified	**Speed: (1) −2%; (2) −12%; (3) −19% **Stride length: (1) −1%; (2) −6%; (3) −10% DLS: (1) −4%; (2) −9%; (3) −10%	—

Lord et al., 2010 [43] (*n* = 29)	71 (7) Off-med	UPDRS: 39 (15)	MMSE: 27 (3)	Stand from a chair, walk 5–11 m (preferred speed; examined in home & distance varied by home)	Motor: (1) carry tray with 2 beakers of water Cognitive: (2) count auditory tones (3) Motor + Cognitive	“Concentrate equally on walking and task(s)”	Speed: (1) −24%; (2) −14%; (3) −32%	—

Lord et al., 2011 [57] (*n* = 50)	69 (7) Off-med On-med	UPDRS: 35 (9) UPDRS: 23 (9)	MMSE: 28 (2)	Walk 6 m, turn 180°, walk 6 m (examined in home)	Motor: carrying a tray with 2 cups of water	“Concentrate on task as a whole”	Speed: −13% Stride time Variability: −8% Speed: −11% Stride time Variability: −21%	—

O'Shea et al., 2002 [44] (*n* = 15)	68 (7)	Modified Webster Scale:12 (6)	STMS: 30 (3)	Walk 10 m (preferred pace)	Motor: (1) coin transfer Cognitive: (2) serial-3 subtraction	Not specified	Speed: (1) −18%; (2) −19% Stride length: (1) −14%; (2) −12% DLS: (1) −3%; (2) −6%	Motor: (1) −17.4% Cognitive: (2) −5%

Plotnik et al., 2009 [48] (*n* = 21)	72 (7)	UPDRS: 20 (8)	MMSE: 28 (1)	Walk 2 min in a level, 25 m corridor (comfortable pace)	Cognitive: serial-7 subtraction	No prioritization	Phase coordination index: −47%	—

Plotnik et al., 2011 [45] (*n* = 30)	66 (7)	UPDRS: 35 (10) H&Y: 2.1 (0.6)	MMSE: 29 (1)	Walk ~80 m in a level, ~20 m corridor (comfortable pace)	Cognitive: (1) serial-3 subtraction; (2) serial-7 subtraction	Not specified	Speed: (1) −17%; (2) −23% Stride length: (1) −11%; (2) −15% Stride time variability: (1) −39%; (2) −51%	—

Rochester et al., 2004 [46](*n* = 20)	65 (8)	H&Y: 2.7 (0.7)	MMSE: 27 (2)	Stand from a chair, walk 6.6 (1.5) m, return (preferred speed, examined in home & distance varied by home)	Motor: (1) carrying tray with 2 cups of water; Cognitive: (2) autobiographical memory task;(3) Motor + Cognitive	Not specified	Speed: (1) −9%; (2) −21%; (3) −23% Step length: (1) −9%; (2) −16%; (3) −21%	—

Rochester et al., 2008 [58] (*n* = 130)	67 (8)	UPDRS: 33 (11)	MMSE: 28 (2)	Walk 6 m, turn 180°, walk 6 m (preferred pace)	Motor: carrying a tray with 2 cups of water	“Concentrate equally on all tasks”	Speed: −13%	—

Spildooren et al., 2010 [59]								
Freezers (*n* = 14)	69 (7) Off-med	UPDRS: 38 (14) H&Y: 2.5 (0.5)	MMSE: 28 (1)	Walk 5 m: (1) straight; (2) turn 180°; (3) turn 360°	Cognitive: color identification (auditory attentional task)	No prioritization	Steps: (1) −25%; (2) −16%; (3) −13% Time: (1) −23%; (2) −13%; (3) −10%	—
Non-freezers (*n* = 14)	67 (7) Off-med	UPDRS: 34 (10) H&Y: 2.4 (0.3)	MMSE: 29 (1)				Steps: (1) −7%; (2) +1%; (3) −2% Time: (1) −9%; (2) −1%; (3) −3%	—

Yogev et al., 2005 [50] (*n* = 30)	71 (8)	UPDRS: 18 (8) H&Y: 2.3 (0.4)	MMSE: 28 (2)	Walk 2 min in a level, 25 m corridor (comfortable pace)	Cognitive: (1) listen to a tape & answer questions; (2) above task + phoneme monitoring; (3) serial-7 subtraction	No prioritization	Speed: (1) −10%; (2) −13%; (3) −19%Stride time variability: (1) −1%; (2) −6%; (3) −27%	Cognitive: (1) −42%

Yogev et al., 2007 [47] (*n* = 21)	72 (7)	UPDRS: 20 (8) H&Y: range 2-3	MMSE: 28 (1)	Walk 2 min in a level, 25 m corridor(comfortable pace)	Cognitive: serial-7 subtraction	No prioritization	Gait asymmetry: −43%	—

*: data collapsed across forward & backward walking; **: data collapsed across PD & control groups; Cog.: cognitive status; DLS: double limb support; DRS-2: Dementia Rating Scale-2; DTC: dual-task cost; H&Y: Hoehn & Yahr stage; Instruct.: instructions provided during dual-task conditions; MMSE: Mini-Mental State Examination; MoCA: Montreal Cognitive Assessment; STMS: Short Test of Mental Status; Steps: number of steps required to complete walking task; Time: time required to complete walking task; UPDRS: Unified Parkinson Disease Rating Scale, motor examination.
